# Localizing Spectral Interactions in the Resting State Network Using the Hilbert–Huang Transform

**DOI:** 10.3390/brainsci12020140

**Published:** 2022-01-21

**Authors:** Ai-Ling Hsu, Chia-Wei Li, Pengmin Qin, Men-Tzung Lo, Changwei W. Wu

**Affiliations:** 1 Bachelor Program in Artificial Intelligence, Chang Gung University, Taoyuan 33305, Taiwan; alhsu@mail.cgu.edu.tw; 2 Department of Psychiatry, Chang Gung Memorial Hospital at Linkou, Taoyuan 33305, Taiwan; 3 Department of Radiology, Wan Fang Hospital, Taipei Medical University, Taipei 11696, Taiwan; 799032@w.tmu.edu.tw; 4 Key Laboratory of Brain, Cognition and Education Sciences (South China Normal University, Ministry of Education), Center for Studies of Psychological Application and Guangdong Key Laboratory of Mental Health and Cognitive Science, South China Normal University, Guangzhou 510631, China; qin.pengmin@gmail.com; 5 Pazhou Lab, Guangzhou 510335, China; 6 School of Psychology, South China Normal University, Guangzhou 510631, China; 7 Department of Biomedical Sciences and Engineering, National Central University, Taoyuan 32049, Taiwan; mzlo@ncu.edu.tw; 8 Graduate Institute of Mind Brain and Consciousness, Taipei Medical University, Taipei 11031, Taiwan; 9 Brain and Consciousness Research Center, Shuang Ho Hospital-Taipei Medical University, New Taipei 23561, Taiwan

**Keywords:** resting-state fMRI, ensemble spectral interaction, Hilbert–Huang transform, amplitude-to-amplitude coupling, time-frequency map, wavelet analysis

## Abstract

Brain synchronizations are orchestrated from neuronal oscillations through frequency interactions, such as the alpha rhythm during relaxation. Nevertheless, how the intrinsic interaction forges functional integrity across brain segregations remains elusive, thereby motivating recent studies to localize frequency interactions of resting-state fMRI (rs-fMRI). To this point, we aim to unveil the fMRI-based spectral interactions using the time-frequency (TF) analysis; however, Fourier-based TF analyses impose restrictions on revealing frequency interactions given the limited time points in fMRI signals. Instead of using the Fourier-based wavelet analysis to identify the fMRI frequency of interests, we employed the Hilbert–Huang transform (HHT) for probing the specific frequency contribution to the functional integration, called ensemble spectral interaction (ESI). By simulating data with time-variant frequency changes, we demonstrated the Hilbert TF maps with high spectro-temporal resolution and full accessibility in comparison with the wavelet TF maps. By detecting amplitude-to-amplitude frequency couplings (AAC) across brain regions, we elucidated the ESI disparity between the eye-closed (EC) and eye-open (EO) conditions in rs-fMRI. In the visual network, the strength of the spectral interaction within 0.03–0.04 Hz was amplified in EC compared with that in EO condition, whereas a canonical connectivity analysis did not present differences between conditions. Collectively, leveraging from the instantaneous frequency of HHT, we firstly addressed the ESI technique to map the fMRI-based functional connectivity in a brand-new AAC perspective. The ESI possesses potential in elucidating the functional connectivity at specific frequency bins, thereby providing additional diagnostic merits for future clinical neuroscience.

## 1. Introduction

The importance of brain science lies in the time-variant synchronization and desynchronization of neuron assemblies across space, forming dynamic regional oscillations over a wide range of frequency complexes. To date, several academic studies have discovered the importance of neural oscillations in daily life by demonstrating associations between electrophysiological components (e.g., local field potential, LFP) on the basis of multi-type physiological states or cognitive processes. For example, a strong alpha-band (8–13 Hz) activity in electroencephalography (EEG) emerges during wakeful relaxation when the eyes are closed, whereas a gamma-band (>30 Hz) activity is associated with the genesis of behavior [1,2]. Conventionally, the majority of research articles on electrophysiological signals have focused on the correspondence between the power of high-frequency oscillations and the performances of task engagements. Alternatively, a recent perspective has attracted public attention to endogenous long-range synchronizations in neural systems, particularly in low-frequency oscillations. Studies have indicated that infra-slow (0.01–0.10 Hz) electrophysiological fluctuations reflect the integrations within concurrently active neuronal communities [3], plausibly associating with the neuroplasticity processes such as memory consolidation [4]. By combining the electrophysiological measure and functional magnetic resonance imaging (fMRI) signal in the resting state [5], Wilson et al. demonstrated that the connectivity strength of the LFP signal is frequency-dependent and differs across regions within the local circuits of the primary somatosensory cortex; furthermore, the interregional connectivity of the resting-state fMRI (rs-fMRI) covaries with that of LFPs in various frequency bands, supporting the neural basis of rs-fMRI [6]. Therefore, the frequency characteristics of rs-fMRI signals, stemming from hemodynamic mechanisms, presumably preserve the underlying physiology of the neural oscillations and network integrations within the infra-slow frequency range [7,8,9].

Specific oscillation frequencies possess either physiological or cognitive inferences, and the emerging evidence indicates that cross-frequency (spectral) interactions between neuronal oscillations associate with distinct emotional states and cognitive processes [10,11]. For example, the spectral interaction between theta and gamma frequency oscillations occurs in the rodent hippocampus [12]. This spectral interaction is suggested to coordinate communication between distinct areas and is associated with memory processes and performance. Several other electrophysiological studies have demonstrated that the spectral interaction between distinct neural oscillations occur during visual and auditory tasks [13], and the interaction strength reflects the working memory maintenance of multiple items [14]. Nevertheless, the limited spatial information provided by electrophysiological signals constrain further discovery on both cortical and subcortical brain systems. Alternatively, on the basis of the hemodynamic perspective, rs-fMRI studies presented the spectral interactions with specific spatial configurations [15], which related to the network properties of the large-scale functional connectome [7]. Previous study showed the association between low-frequency rs-fMRI signals (<0.1 Hz) and EEG rhythms (Delta, Theta, Alpha, Beta, and Gamma) and found the positive Beta power correlation with the rs-fMRI signal in the default-mode network (DMN) and the negative Alpha correlation with the rs-fMRI signal in the visual network [16]. Chang et al. also presented that EEG alpha power shows an inverse relationship with the connectivity strength of DMN and dorsal attention network [17]. The fMRI spectrum contains promising components that constitute the brain-network connectivity, especially in the 0.02, 0.04, and 0.08 Hz, where every brain network contains its own frequency specificity [7,18]. The evidence supports that the specific fMRI frequency information could provide promising neurophysiological information underlying sleep and consciousness [19]. Additionally, such resting-state functional connectivity showed its clinical potential by revealing the network differences in neurological and psychiatric patients within and between different frequency bands [20].

Since taking both temporal and spectral information into consideration could further reveal the underlying neurophysiological information of resting-state functional connectivity, one novel approach to achieve this goal is to perform the time-frequency (TF) analysis prior to the functional connectivity analysis. However, using the TF maps for functional connectivity analysis is not adopted in the rs-fMRI field because of the following two reasons: (1) compared with the broadband EEG signal spectrum (1–70 Hz), the fMRI hemodynamic responses contain only the ultra-low-frequency spectrum (usually <0.25 Hz with 2-s repetition time); and (2) Fourier-based TF analyses are subject to the uncertainty principle. For the former part, indeed the low sampling rate of the fMRI signal constrains its frequency range within a low-frequency range technically, but the slow hemodynamic response function (HRF) automatically transforms the high-frequency neural oscillations into the low-frequency domain. Therefore, a series of studies reported that the low-frequency fMRI signal correlates with EEG signals across different frequency bands. Furthermore, Thompson et al. identified the specific frequency corresponding to brain connectivity efficiency [7], and Yaesoubi et al. also suggested different connectivity patterns corresponding to distinct frequency states in the normal human brain [21]. However, the spectral information has not been widely applied to the clinical neuroscience because of its technical obstacle. Second, the commonly adopted Fourier-based TF analyses (i.e., the short-time Fourier transform (STFT) or wavelet-based TF maps [7,22] are constrained by the constant product between time and frequency (the uncertainty principle) [23], where the selection of window length in the time domain determines the spectral resolution in the frequency domain. Henceforth, the pre-defined fixed time window (STFT) leads to the point spread function (PSF) broadening in the frequency domain, and the variant time window (wavelet) presents limited accessibility within the cone of influence (COI) in the resulting TF maps [15]. Relatively, the Hilbert–Huang transform (HHT), unrestricted by the uncertainty principle, empowers the calculation of instantaneous frequency of the intrinsic signal components [24,25]. In other words, HHT can be used to carry out the TF maps with characteristics of high resolution and full accessibility, facilitating future fMRI-based functional connectivity analyses with spectral interactions. 

Therefore, for disclosing the high-resolution TF map of the rs-fMRI signal, we employed the HHT in this study to circumvent the restriction of the uncertainty principle. We applied the HHT to elucidate spectral interactions of the rs-fMRI signal on the well-addressed eye-closed (EC)/eye-open (EO) conditions. We hypothesized that the rs-fMRI signals in the visual network changed their frequency coupling between the EC and EO conditions. This statement holds true for the elevated alpha rhythm in the EC condition, which enables changes in spectral interactions derived from the fMRI TF maps. In this study, we first demonstrated that the resolution of the TF map generated using HHT outperforms that generated using wavelet transform. Next, we demonstrated the characteristics of spectral interaction with both the simulated and real fMRI data. Finally, we characterized the spectral interactions on the basis of HHT TF maps to detect conditional changes in frequency bands within the visual network. With the advantages of the high-resolution TF map, fMRI techniques have the potential to reveal cognitive functionality through spectral interactions within specific spatial locations in the brain.

## 2. Materials and Methods

### 2.1. Image Acquisition

Fifteen right-handed, healthy volunteers (age, 32.6 ± 10.1 y; female/male, 8/7) were enrolled in this study with the approval of the Institutional Review Board of Taipei Medical University (TMU-JIRB 201411043). The dataset was the subset of the normal control data in this clinical protocol and reused for technical development in this study. Informed consent was obtained from all subjects involved in the study prior to the examinations. Data were acquired with a 3 Tesla clinical scanner (Discovery MR750, GE Healthcare, Milwaukee, WI, USA) by using a standard eight-channel head coil for signal detection and a whole-body coil for radiofrequency excitation. A high-resolution T1-weighted structural image was acquired using a 3D fast spoiled gradient-echo sequence (repetition time (TR)/echo time (TE) = 8.2/3.2 ms; flip angle = 8°; 193 slices with 1 × 0.88 × 0.88 mm^3^ voxels without inter-slice gap]. To analyze the spectral interaction changes between the two conditions (EO/EC), a single-shot gradient-echo echo planar imaging (GE-EPI) sequence was adopted to acquire rs-fMRI data by using the following parameters: TR/TE = 2000/30 ms; flip angle = 90°; FoV = 220 mm; matrix size = 64 × 64; slice thickness = 3.4 mm; slice gap = 0 mm; 37 slices in total. Each EPI session contained 240 time points, equivalent to 8 min in acquisition. The scanning order of the EC/EO conditions was counterbalanced to reduce the systematic bias in rs-fMRI sessions. During the rs-fMRI sessions, the lights in the scanner room were turned off, and the participants were instructed to relax, stay awake, and not focus on anything in particular. Specifically, in the EC session, a black screen was presented to ensure that participants were exposed to dim light; they were instructed to keep their eyes closed throughout the scanning. In the EO session, a gray screen was presented; the participants were instructed to keep their eyes open and to look towards the screen. After scanning, participants were asked whether they had remained awake; none reported falling asleep.

### 2.2. fMRI Preprocessing

All scanning images were pre-processed using AFNI [26], FSL [27], and SPM8 (Welcome Department of Cognitive Neurology, Institute of Neurology, London, UK). The dataset underwent the following preprocessing steps: removal of the first ten time points for intensity stability, motion correction on the remaining 230 time points, detrending, despiking, spatial smoothing to 5-mm full width at half maximum (FWHM), and regressing out identifiable noise components, including both white matter (WM) and cerebrospinal fluid (CSF) [28]. Motion correction was performed using FSL (FLIRT) to reduce possible head movements for each rs-fMRI time series. The spike and estimated polynomial trends were removed from the time series because of hardware instability. In the preprocessing of the rs-fMRI signal, spatial smoothing is a crucial factor that affects the temporal signal-to-noise ratio [29], physiological contributions in spontaneous oscillations [30], and connectivity strength [31]. Thus, spatial autocorrelation was specifically controlled to 5 mm FWHM by using 3dBlurToFWHM [26]. Furthermore, to prevent the additional smoothing induced by spatial normalization [31], all functional images were analyzed in the native space in both preprocessing and the further analysis. Eight noise components modeled by six motion parameters and the mean time series of WM and CSF were then regressed out using the general linear model. To optimally generate individual WM and CSF masks, we implemented the following three processing steps: (1) T1w image segmentation was conducted for WM and CSF probability maps, and initial WM/CSF masks were created by setting a probability threshold of 50% and were then transformed to match the rs-fMRI resolutions. (2) An additional prior CSF mask was generated in MNI space by selecting both the third and fourth ventricles of the built-in CSF map in the SPM8 toolbox with a probability threshold of 35%, and the prior WM mask was generated by using the SPM WM template with a probability threshold of 50%. These processed prior masks were then inverse-normalized to the individual EPI space by using an inverse normalization matrix. (3) The final individual WM/CSF masks were then generated from the conjunction areas of the initial masks of (1) and the prior masks of (2). 

### 2.3. Time–Frequency Spectrum Using the Hilbert–Huang Transform

To successfully access the information on spectral interactions (cross-frequency coupling) from the fMRI signals with limited time points, an alternative approach is required to overcome the stationarity assumption and uncertainty principle inherited from the Fourier transform. Therefore, we implemented the HHT to extract physiological dynamics without the constraints of stationarity and linearity on the rs-fMRI signal. Unlike the Fourier transform, which uses the given mathematical basis, the HHT [24] method adaptively decomposes the input signal into a set of intrinsic mode functions (IMFs), calculates the instantaneous frequency of IMFs, and then illuminates the spectral details in a Hilbert-based TF map, where the IMFs preserve the physical meaning of the original dataset. By using this strategy, either amplitude-modulated or frequency-modulated nonlinear signals can be highlighted in a fine resolution TF map without stationarity and linearity constraints. Specifically, signal decomposition is processed using the method of noised-enhance ensemble empirical mode decomposition (EEMD) through the following steps: (1) addition of a white noise at a specific level to the signal, (2) decomposition of the signal with noise, and (3) repeated execution of (1) and (2) and averaging of the IMFs of all repetitions. As the number of repetitions increased, the resulting IMFs demonstrated their potential to reflect the true fluctuations in the fMRI data with a high frequency sensitivity [32,33]. To delineate the dynamic characteristic of raw-amplitude data in terms of spectral amplitude, the Hilbert spectrum of each IMF was summated to yield a TF map. The spectral amplitude here denotes the envelope amplitude of a certain frequency carrier rather than the raw amplitude of carrier fluctuations, which is the major difference between the current method and the canonical seed-correlation analysis in rs-fMRI. Furthermore, the spectral amplitude in the TF map can reflect the dynamic variations of a certain frequency or even when a frequency shift occurs at a certain period of time.

Technically, for mitigating the potential end effects and aliasing artifacts in Hilbert spectrum [34], the data were extended to three more time points beyond the existing data range by using the mirror method and then interpolated (upsampled) by a factor of four using the spline method prior to the EEMD process. Next, the EEMD was performed with a noise level of 20% and 200 realizations, which has been demonstrated to effectively illustrate the dynamic frequency response in an fMRI signal [32,33]. The resulting IMFs with frequency >0.01 Hz were reserved to generate the Hilbert TF map through Hilbert spectral analysis. The EEMD-aided Hilbert TF map calculation was performed in a voxel-wise manner on the preprocessed fMRI datasets. The extending time points were removed, and only the Hilbert TF map with the existing data range was used for the following analysis. Subsequently, to increase the spectrum signal-to-noise ratio, we calculated the average of the Hilbert TF maps across voxels within the regions of interests (ROIs). By focusing on the representatives in the visual network, the ROIs defined by the scheme of a large-scale network [35], which integrates the fMRI meta-analysis findings in the MNI coordinates, were used in the current study. The ROIs within the visual network were generated using the given coordinates within a 5-mm-radius sphere and were then inversely normalized to the individual space. 

### 2.4. Illustrative Example of a Time–Frequency Spectrum and Spectrum Interaction Map

**Figure 1:**
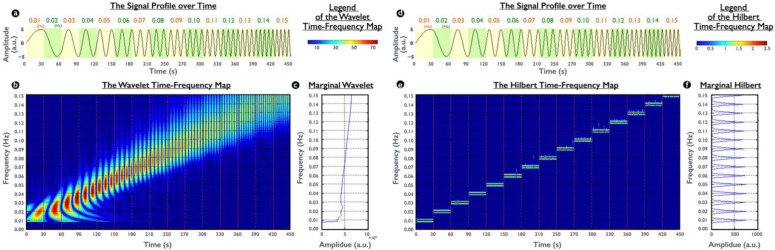
Illustration of time–frequency maps obtained using wavelet analysis and HHT. (**a**) A synthetic time-variant signal with a frequency starting from 0.01 Hz to 0.15 Hz at 0.01 Hz increments for every 30 s. (**b**) Wavelet time–frequency map of (**a**) showing broaden point spread functions (PSF) along the frequency axis. In time–frequency (TF) maps, time is plotted on the *x*-axis and frequency on the *y*-axis. The wavelet TF map and Hilbert TF map were color coded for absolute magnitude and magnitude, respectively. (**c**) The marginal wavelet shows that the signal frequency starts from 0.01 Hz and its energy slightly increases with the frequency increments. (**d**) Same as (**a**). (**e**) Hilbert TF map of (**d**) with the frequency representation bounded in skeleton lines without PSF broadening. (**f**) The marginal Hilbert precisely highlights each frequency without contamination by other frequencies.

An illustrative comparison between the wavelet TF map and Hilbert TF map on a synthetic signal is presented in Figure 1. The wavelet analysis was applied using six cycles of the Morlet wavelet with a length of 5 standard deviations of the Gaussian kernel in this study. The sinusoidal signal comprises of 15 frequency components, starting at 0.01 Hz with a 0.01 Hz increment every 30 s. This frequency range of the simulation was selected to represent the frequency characteristic of the fMRI hemodynamic responses. For visualization purposes, the Hilbert TF map present here was smoothed using a Gaussian low-pass filter. In addition, to further reveal the difference between two transformations on a frequency resolution, the marginal wavelet and marginal Hilbert were calculated through a summation of the total amplitude over time from the wavelet and Hilbert TF maps, respectively. Because the wavelet is subject to the uncertainty principle in a Fourier basis, the marginal wavelet exhibited smoothed contours without detailed frequency specificity; by contrast, by using the adaptive Hilbert basis, frequency variation in the marginal Hilbert was bounded in multiple peaks, reflecting the real condition of the simulated signal. 

**Figure 2:**
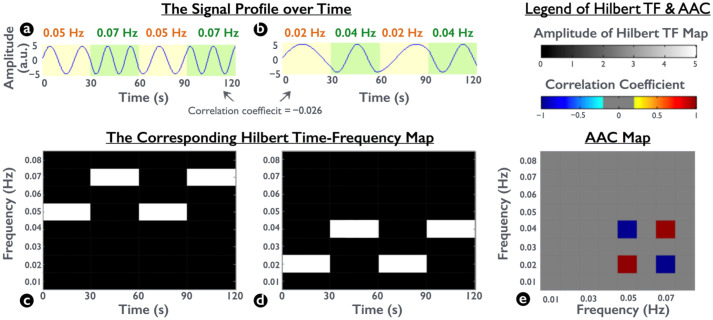
Demonstration of the amplitude–amplitude coupling (AAC) between two frequency-modulated signals. (**a**,**b**) Synthetic signals that exhibited a change in frequency every 30 s with two frequency oscillations. Pearson correlation between the two signals is −0.026. (**c**,**d**) Hilbert TF maps of the signal in (**a**,**b**), respectively. (**e**) Correlation for a pair of two HTF time series at any two frequencies, yielding the AAC map. The positive correlation appears at the coordinate 0.05 × 0.02 Hz because of the same temporal dynamic patterns at 0.05 Hz in (**c**) and those at 0.02 Hz in (**d**); similarity, the AAC map depicts a negative correlation at the coordinate 0.05 × 0.04 Hz because of the opposite patterns between 0.05 Hz in (**c**) and 0.04 Hz in (**d**).

To demonstrate spectral interactions, we simulated two synthetic frequency-modulated waveforms. In each waveform, the signals changed frequency every 30 s twice within the 2-min duration. One signal was composed of 0.05-Hz and 0.07-Hz oscillations (Figure 2a), and the other was composed of 0.02-Hz and 0.04-Hz oscillations (Figure 2b). To access the spectral interaction between the two simulated waveforms, we calculated their corresponding Hilbert TF maps and then performed a Pearson correlation between two Hilbert TF maps by collapsing the temporal information. This approach enabled the coherence measurement of spectral amplitudes at any frequency pair for the amplitude–amplitude coupling (AAC) map (Figure 2e) [33,36,37]. 

### 2.5. Spectral Interaction within a Visual Network 

To globally elucidate the characteristic of spectral interactions within the visual network, for each participant, we first selected 5 out of 30 ROIs from the visual network to generate a representative Hilbert TF map and then selected another 5 to generate another representative. Next, the aforementioned correlation analysis was conducted between the continuous temporal dynamics of the two representatives, resulting in an AAC map within the visual network. Moreover, to highlight the network changes between the EC/EO conditions and to reduce the variance of AAC maps, we randomly sampled the ROI subsets in the visual network ten times and averaged the findings to generate AAC maps, and then converted the values to Fisher z values to yield the ensemble spectral interaction (ESI) maps for each visual condition.

### 2.6. Nonparametric Statistical Testing

To more accurately locate frequency-band alterations elicited by the condition changes, we applied a non-parametric permutation *t* test with a paired sample design on ESI maps between the EC and EO conditions, and then corrected for multiple comparisons using the Bonferroni approach. Specifically, the ESI maps were first regridded to a size of 0.01 Hz by averaging their values per grid (resulting in 7 × 7 grids). Second, within each pair, the labels of the EC/EO conditions were randomly relabeled (swapped) to the regridded ESI maps of each individual. Third, a paired *t* test between the relabeled EC/EO ESI maps was performed to obtain a permutated *t* map. Forth, we performed five thousand permutations to create the permutated *t* distribution of the relabeled ESI maps between two conditions. Finally, the *t* map with the actual labeling between visual conditions was considered statistically significant if the two-tailed probability of the true *t* value in the permutated *t* distribution was higher than 97.5% or was lower than 2.5% (*p* < 0.05, Bonferroni corrected for the 49 comparisons). In contrast to ESI, we further examined the between-condition connectivity difference by using the canonical functional connectivity analysis (Pearson correlation) [5,6], calculated from both a broadband or a bandpassed raw-amplitude fMRI signal (0.01–0.08 Hz bandpass filter and 0.03–0.04 Hz bandpass filters, respectively).

## 3. Results

Figure 2 presents a plot of the two simulated raw-amplitude waveforms (Figure 2a,b) and the correlation of two Hilbert TF maps (Figure 2c,d). Although the raw-amplitude correlation between Figure 2a,b revealed miniature synchronization (−0.026) by using Pearson correlation analysis, their time-variant spectral amplitudes were identical (e.g., 0.05 Hz in Figure 2a vs. 0.02 Hz in Figure 2b, also known as frequency modulation). Therefore, the corresponding spectral-amplitude correlations at the four frequency coordinates were at the highest level (1 or −1). Because the simulated signals depicted the relationship between frequency modulations, the AAC map was expected to identify spectral interactions between the two Hilbert TF maps.

**Figure 3:**
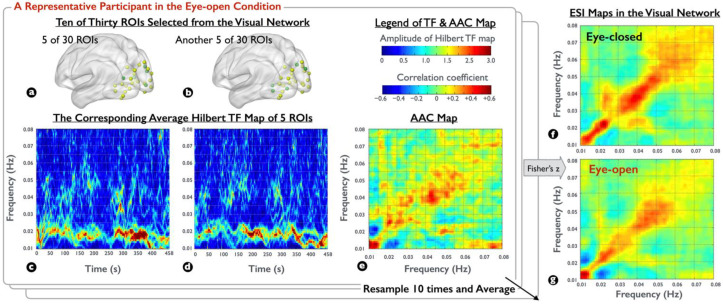
Demonstration of the ESI maps on participant-level rs-fMRI signals. (**a**,**b**) Of 30 ROIs, 5 were selected from the visual network for generating a representative Hilbert TF map. (**c**,**d**) Averaged Hilbert TF maps in the EO condition for the selected 5 ROIs in (**a**) and another 5 selected ROIs in (**b**), respectively. (**e**) AAC map derived from the correlation between each frequency pair of (**c**,**d**) by collapsing the temporal information. (**f**,**g**) ESI maps in the EC and EO conditions obtained by averaging AAC maps from ten times ROI resampling procedure and then applying Fisher’s z-transform.

To examine the difference in spectral interactions between the EC and EO conditions in the visual network, we generated individual ESI maps in each resting-state condition. Figure 3 illustrates the process of ESI maps obtained from the two visual conditions for a representative participant, with three observations. First, the spectral amplitude of Hilbert TF maps during the EO condition (Figure 3c,d from two selected ROI sets) was stronger in the low-frequency range (0.01–0.03 Hz) than that in the high-frequency range (0.03–0.06 Hz), fulfilling the power law in physiological signals. Second, the spectral-amplitude pattern was continuous in the low-frequency range but was intermittent in the high-frequency range. Third, despite the cross-frequency discrepancy in spectral amplitudes, the AAC map in Figure 3e, similar to Figure 2e, was strong in the high-frequency range, clearly indicating the frequency selectivity in spectral interactions. After the ensemble average of different ROI sets and Fisher’s z-transform, Figure 3f,g denote the ESI maps corresponding to the EC and EO conditions, respectively. Through visual inspection, the coupling strength within the specific frequency band (0.03–0.04 Hz and 0.06–0.08 Hz) appeared to be amplified in the EC condition for the representative participant, as well as the spectral interaction in the cross-frequency region (between 0.01–0.02 and 0.02–0.03 Hz). 

**Figure 4:**
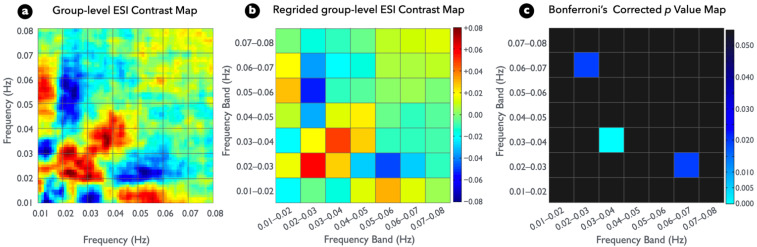
Significant differences among participant ESI between visual conditions (*p* < 0.05, two tailed, Bonferroni corrected). (**a**) Group differences in the ESI map for the EC versus EO comparison. (**b**) Same as (**a**), but at the regridded level. (**c**) Nonparametric statistical *p* value map with Bonferroni correction for examining group ESI difference.

Figure 4a presents the group-level ESI contrast map between the two conditions. To fit the typical frequency bins of rs-fMRI (0.01 Hz) and to control the false positive rate from multiple comparisons of statistical testing, the plot in Figure 4a was regridded to a bandwidth of 0.01 Hz, and the result was displayed as Figure 4b. A permutated paired *t* test (EC-EO contrast) resulted in significantly higher spectral interactions within the frequency band of 0.03–0.04 Hz in the regridded ESI maps; however, it resulted in significantly lower cross-frequency interactions between frequency bands of 0.02–0.03 and 0.06–0.07 Hz (Figure 4c) (see Figure S1 for detailed information). Moreover, Figure 4b presents a strong but insignificant conditional difference within the frequency band of 0.02–0.03 Hz because of the large between-subject variability.

**Figure 5:**
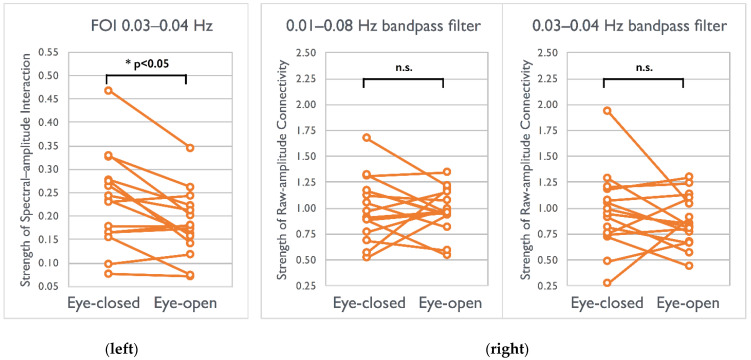
Difference in the strength of spectral–amplitude interaction (**left**) and raw-amplitude connectivity (**right**) between visual conditions across 15 participants. (**left**) The strength of ESI in the EC condition was higher than that in the EO condition (* *p* < 0.05, two tailed, Bonferroni corrected). (**right**) Nonsignificant (n.s.) connectivity difference between conditions for two types of filtered signal (0.01–0.08 Hz bandpass filter and 0.03–0.04 Hz bandpass filters). FOI: frequency of interest.

After localizing spectral interactions significantly differed between the EC–EO conditions, we further examined the strength of canonical functional connectivity calculated using filtered raw fMRI signals for comparison. Signals filtered by 0.01–0.08 Hz (*p* > 0.35) or those filtered by 0.03–0.04 Hz did not exhibit between-condition differences (*p* > 0.19) within the visual network (Figure 5). Only ESI within the specific frequency of interest (FOI) yielded a high sensitivity in detecting elevated synchronizations in the EC condition within visual networks.

## 4. Discussion

In the current study, we applied HHT to compare the disparity of spectral interactions between the EC/EO conditions in rs-fMRI signals. We successfully observed the amplification of spectral interactions in the EC condition within a certain frequency band (0.03–0.04 Hz) in the visual network. This observation proved the efficacy of this innovative method in localizing the spectral interactions given a pair of synchronous oscillatory signals. We performed the ESI using the following three steps: (1) decomposing fMRI signals through noise-enhanced EEMD, (2) conducting Hilbert spectral analysis to obtain TF maps, and then (3) conducting cross-correlation and collapsing the temporal information of the TF maps for obtaining spectral interactions at each frequency. Through this strategy, the aforementioned limitation for studying spectral interactions was completely prevented. First, by using the Hilbert TF map, we successfully disclosed that a certain fMRI spectrum (0.03–0.04 Hz) within 0.01–0.08 Hz contributed to the connectivity changes between the EC and EO conditions. Second, the ESI can be accomplished only because the Hilbert TF map provides spectral features without the PSF broadening and the COI restriction. Third, although ESI is a stationary index for frequency modulation (because of correlation analysis), the Hilbert TF map disclosed the dynamic changes with full accessibility, revealing nonstationary TF information for further dynamicity studies. Most importantly, because HHT considers frequency and amplitude as functions of time separately without the constraint of the Fourier-based uncertainty principle, the Hilbert TF map does not possess the cone of influence (COI) limit in wavelet TF maps, which makes the implementation of ESI maps possible (see Figure S2). The other popular dynamic analysis method is the use of short-time Fourier transform (STFT), which yields frequency spectra by setting a small duration in the temporal domain (window size). Although this method does not present the COI problem, the selection of window size in STFT would determine both temporal or spectral resolutions at the same time (uncertainty principle). With limited time points in each fMRI session, the resulting fMRI-based TF map would possess general issues of PSF broadening in the frequency domain [38]. For example, applying the STFT to the current dataset with a window size of 48-s shall provide a TF map with a frequency resolution of 0.01 Hz but with only 10 time points, sacrificing the statistical power in connectivity analysis. Furthermore, Keilholz et al. indicated an issue in STFT, that a predefined window size determines the frequency of interests, which may alter the polarity of the connectivity strengths [39]. Yeh et al. indicated that inappropriate selection of the window size could lead to spurious spectral interactions [33]. In terms of brain connectivity, Hindriks et al. reported the correlation with predefined window lengths is almost impossible to correctly detect the dynamic changes of functional connectivity [40]. Therefore, according to our review of the relevant literature, the HHT-based ESI method implemented in this study is suitable for elucidating the cross-frequency coupling in fMRI signals at the current stage. Given any pair of functional profiles, ESI maps can be applied to the frequency coupling between regions or between brain networks. In addition, although ESI maps were originally used for yielding spectral interactions in fMRI signals, this novel method is applicable to other functional imaging modalities, such as EEG, magnetoencephalography, or positron emission tomography, for studying spectral interactions from different physiological mechanisms. 

### 4.1. Interpreting Spectral Interaction in an rs-fMRI Signal

Rs-fMRI fluctuations possess specific frequency features with neurophysiological mechanisms, which are the major objective of obtaining the spectral interaction map under low sampling rates and insufficient time points. Spectral interaction not only resembles the concept of functional connectivity by incorporating time curves from two regions to evaluate their mutual relation, but also provides frequency details. ESI maps extract the frequency information on the basis of high-resolution TF maps obtained using Hilbert-spectrum analysis. By collapsing the timing information of two simultaneously recorded signals, ESI maps denote the detailed correlation in the frequency domain. This is possible on the basis of Hilbert transform, because the Fourier-based methods (e.g., STFT, wavelet, or Gabor–Wigner transform) are affected by the uncertainty principle, particularly vulnerable in low-frequency components. However, two intrinsic properties of the ESI method shall be noticed for further applications. First, the Hilbert spectrum outputs the envelope amplitude for every frequency carrier in the TF map, instead of the raw amplitudes in the time curves. Therefore, unlike conventional correlation analyses for counting carrier fluctuations, the ESI map represents the correlation between enveloped amplitudes irrespective of the carrier frequency. Therefore, the ESI map denotes the AAC across different frequency carriers, which was also known as the frequency modulation in the communication principle [33] (also shown in Figure 2). Second, because of collapsing the temporal information, the resulting ESI map is a stationary measure, quantifying the coupling level between envelope amplitudes of a pair of oscillatory waveforms. Therefore, the ESI map is effective for comparison between stationary conditions, such as the cross-sectional differences between the healthy control and patients and longitudinal changes between wakefulness and sleep. In terms of nonstationarity, dynamic analysis based on the Hilbert TF map should be conducted after localizing the influential frequency carrier in the ESI map. Notably, the resampling (ensemble) technique applied in this study was required to enhance the sensitivity of the estimate AAC characteristics for the entire brain network and to bypass the bias of the partial visual network through manual ROI selection, which would be a suggestive method for the ESI calculations.

### 4.2. Frequency Implication in rs-fMRI EC/EO Datasets

When we close our eyes, a series of physiological phenomena occurs: a high density of alpha waves emerges from the synchronizations of neuronal populations and becomes detectable in the occipital lobes [41]; subsequently, the baseline cerebral blood flow decreases [42], which leads to a decreased BOLD-fMRI signal [43]. In the current study, we presented the fMRI spectral contrasts between the EC/EO conditions by the ESI maps without imposing the linearity assumption between the neural activities and fMRI signals. By observing the frequency changes within the visual network, we found the enhanced spectral interaction (correlation) of 0.03–0.04 Hz in the EC condition. Physiologically, the elevated alpha power in the EC condition indicates a large-scale occipital neural synchronization during cognitive disengagements [41], suggesting the reinforcement of internal integrity in the visual network and resulting in the enhanced spectral interaction of 0.03–0.04 Hz in fMRI fluctuations. However, this enhanced functional connectivity was not explicit when either broadband (0.01–0.08 Hz) or bandpassed (0.03–0.04 Hz) fMRI signals were inputted for analysis (shown in Figure 5). This finding is in good agreement with the study by Patriat et al., in which a non-significant but slightly higher EC connectivity was reported within the visual network than the EO connectivity (0.86 vs. 0.81 for intrasession and 0.88 vs. 0.86 for intersession) [44]. Furthermore, because the EEG alpha band is substantially higher than the resulting 0.03–0.04 Hz in the fMRI signal, the exact physiological coupling between these two measurements must be investigated in further studies. Although the physiological mechanism between electrophysiology and the hemodynamics is beyond the scope of the current study, some previous studies have indicated low-pass filter characteristics of hemodynamic responses and the non-linearity between fMRI signals and EEG rhythms [45]. In addition, the ESI map demonstrated its potential to localize the cross-frequency coupling, and current rs-fMRI datasets evidently presented these results; however, further inferences cannot be drawn without validation. The concurrent electrophysiological–hemodynamics measurements, such as EEG–fMRI, are warranted to reveal and interpret the underlying mechanisms of cross-frequency couplings. 

### 4.3. Issues of HHT Processing

In this study, we implemented the Hilbert spectrum to highlight the time-variant frequency shifting. However, several problems must be addressed when HHT is applied to fMRI signals. First, because of the average process of IMFs in EEMD, one frequency signal may possibly be split into different IMFs, reducing its physiological significance for an individual IMF and thus resulting in misinterpretation of the results [34]. To solve this potential problem, previous studies have performed a recursive combination of split IMFs by using orthogonal checking [32,33]. In this study, although this mode splitting may obscure either physical or physiological meaning, it does not affect the reliability of the resultant Hilbert TF map because Hilbert TF maps include the complete time–frequency information of all IMFs. Second, the insufficient sampling rate results in a distorted signal, introduces artificial amplitude modulation in IMFs, and causes the aliasing artifacts in Hilbert TF maps [46]. To avoid these artifacts, prior to the EEMD process, we interpolated the preprocessed rs-fMRI signal by a factor of four by using the cubic spline method. The selected factor was determined using the simulation results of the generated distorted signal and interpolated by multiple factors, and then the TF maps between the distorted and undistorted signals were compared. Specifically, we generated a chirp-like signal with a sampling rate of 10 Hz (Figure 1); however, we subsequently downsampled the signal to 0.5 Hz to simulate a distorted fMRI waveform. Next, three interpolation factors, 2, 4, and 20, were used to obtain the corresponding TF maps. The aliasing artifacts were alleviated using an interpolation factor larger than four in our simulation results.

### 4.4. Considerations and Limitations

We identified several considerations and limitations in the present study. First, instead of selecting spectral power, we used spectral amplitude for referencing the widely adopted unit in verifying functional characteristics of the cross-frequency coupling [6,11,33,47]. Second, unlike the considerable artifacts due to the non-neural noise in the siding-window analysis, this adverse effect was mitigated using the entire existing time series on the basis of the Hilbert transform [48]. Moreover, we used the pair design of EC–EO conditions in the same group to reduce the cross-sectional difference caused by unmatched physiological noise. In this study, we investigated a new approach for localizing spectral interactions in fMRI signals and used EC–EO conditions as the physiological models in humans. Determining the direct association between electrophysiology and rs-fMRI in cross-frequency spectral interactions is indeed important, but it is beyond the scope of this study. Further simultaneous electrophysiology and fMRI recordings are required to determine the relationship between two concurrent independent measurements. Finally, although the nonparametric statistical mapping used in this study enabled the location of changes in specific frequency bands, advanced statistical approaches are warranted for testing the substantial information obtained from the ESI maps.

## 5. Conclusions

We proposed an innovative HHT-based ESI method for investigating the frequency-specific interaction of rs-fMRI signals between two visual conditions. In the visual network, the strength of frequency coupling within 0.03–0.04 Hz was amplified in the EC condition compared with that in the EO condition. By contrast, asking participants to keep their eyes open resulted in the significantly higher cross-frequency interactions between frequency band of 0.02–0.03 and 0.06–0.07 Hz. By using this method, the rs-fMRI signals were decomposed into Hilbert TF maps, allowing spectral localization of the cross-frequency amplitude modulation over time in fMRI signals. By collapsing the temporal information of any pair of TF maps, the ESI map can be used to investigate the frequency coupling between regions, networks, and imaging modalities to thoroughly understand the functional organizations in the brain.

## Data Availability

Data available on request due to restrictions, e.g., privacy or ethical.

## Supplementary Materials

The following supporting information can be downloaded at: https://www.mdpi.com/article/10.3390/brainsci12020140/s1, Figure S1: The comparison between the Hilbert TF maps and the wavelet TF maps from the representative participant; Figure S2: The nonparametric statistical *p* value map with Bonferroni correction for examining group ESI difference.
