# Supplementary material for: Localizing Spectral Interactions in the Resting State Network Using the Hilbert–Huang Transform
Source: Brain Sci. 2022 Jan 21;12(2):140. doi: 10.3390/brainsci12020140 (PMC8870154; doi:10.3390/brainsci12020140)
Supplement: Supplementary file 1 [file brainsci-12-00140-s001.zip › brainsci-1480875-supplementary.pdf]

Supplementary Information

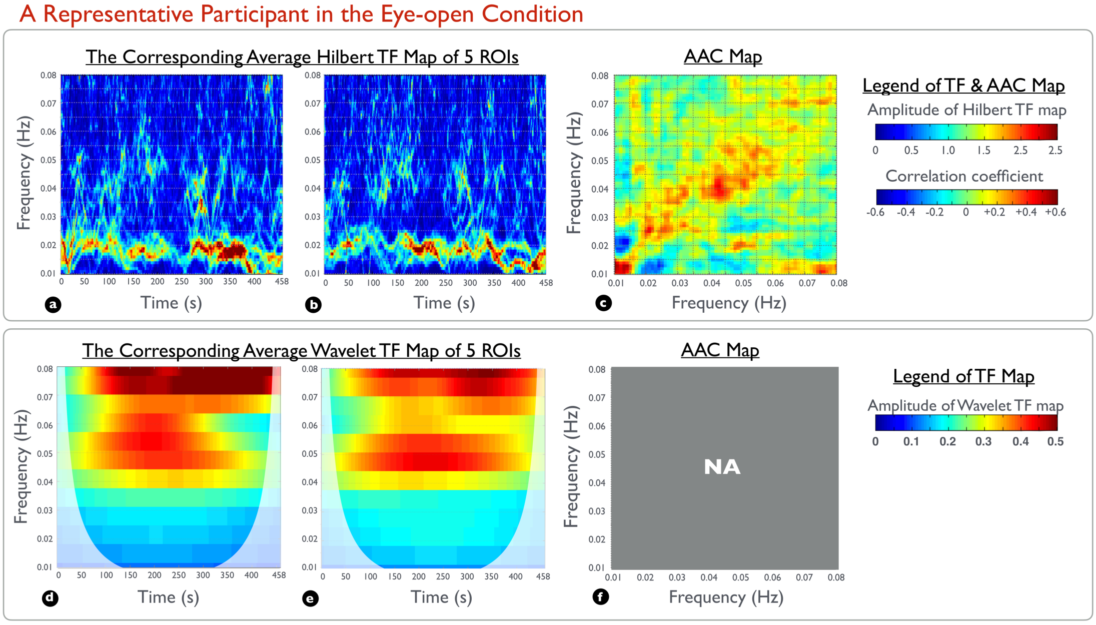

Figure S1. The comparison between the Hilbert TF maps and the wavelet TF maps from the representative participant. (a-c) the Hilbert TF maps and AAC map are identical to Fig. 3c-e. (d-e) the wavelet TF maps corresponds to (a) and (b). Due to the restriction of cone of influence, the AAC map is unachievable using wavelet analysis.

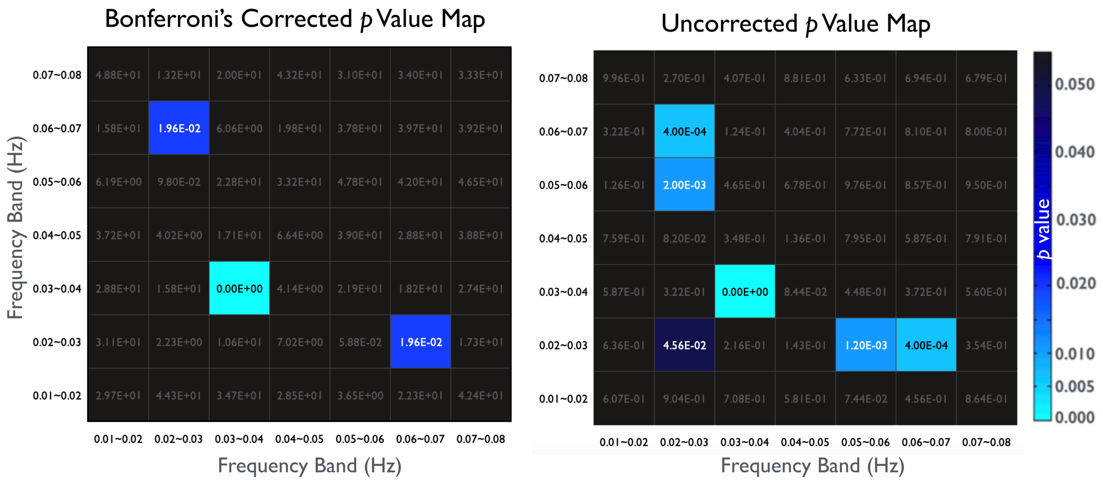

Figure S2 The nonparametric statistical p value map with Bonferroni correction for examining group ESI difference. The left panel shows the corrected p values in the ESI map, as shown in Fig. 4c, and the significant results was color coded; and the right panel shows the uncorrected p values with color-coded significance as well.
